# Modulatory effects of traditional Chinese medicines on gut microbiota and the microbiota-gut-x axis

**DOI:** 10.3389/fphar.2024.1442854

**Published:** 2024-10-09

**Authors:** Tingting Luo, Qingya Che, Ziyi Guo, Tingxia Song, Juanjuan Zhao, Delin Xu

**Affiliations:** ^1^ Department of Medical Instrumental Analysis, Zunyi Medical University, Zunyi, China; ^2^ Department of Cell Biology, Zunyi Medical University, Zunyi, China; ^3^ Department of Immunology, Zunyi Medical University, Zunyi, China

**Keywords:** traditional Chinese medicines, gut microbiota, microbiota-gut-x axis, action mechanism, therapeutic effects

## Abstract

The gut microbiota offers numerous benefits to the human body, including the promotion of nutrient absorption, participation in metabolic processes, and enhancement of immune function. Recent studies have introduced the concept of the gut-organ axis, which encompasses interactions such as the gut-brain axis, gut-liver axis, and gut-lung axis. This concept underscores the complex interplay between gut microbiota and various organs and tissues, including the brain, heart, lungs, liver, kidneys, muscles, and bones. Growing evidence indicates that gut microbiota can influence the onset and progression of multi-organ system diseases through their effects on the gut-organ axis. Traditional Chinese medicine has demonstrated significant efficacy in regulating the gastrointestinal system, leveraging its unique advantages. Considerable advancements have been made in understanding the role of gut microbiota and the gut-organ axis within the mechanisms of action of traditional Chinese medicine. This review aims to elucidate the roles of gut microbiota and the gut-organ axis in human health, explore the potential connections between traditional Chinese medicine and gut microbiota, and examine the therapeutic effects of traditional Chinese medicine on the microbiota-gut-organ axis. Furthermore, the review addresses the limitations and challenges present in current research while proposing potential directions for future investigations in this area.

## 1 Introduction

In recent years, with the deepening understanding of the important role of the gut microbiota (GM) in health and disease, the interaction between traditional Chinese medicine (TCM) and the GM has become a hot research topic. The GM is considered the second genome of the human body and plays a key role in maintaining physiological balance, regulating the immune system, metabolism, and neurological function (Cui et al., 2023; Wei et al., 2024). As a treatment system with a long history, TCM emphasizes the overall concept and individual differences in its theoretical basis, and has the potential to regulate the GM, which provides new ideas for modern medicine. Research shows that active ingredients in TCM can interact with the GM through multiple mechanisms (Gong et al., 2020). For example, TCM ingredients can regulate the composition and function of GM and restore intestinal microecological balance, thereby improving intestinal barrier function and immune response (Yang et al., 2022). In addition, the multi-target and multi-pathway regulatory properties of TCM give it unique advantages in the treatment of intestinal-related diseases (such as inflammatory bowel disease, diabetes, obesity, etc.). By regulating the GM, TCM can affect the host metabolism and immune function, thereby regulating systemic health (Yue et al., 2022).

In recent years, an increasing number of studies have focused on the interaction between bioactive metabolites of TCM and the GM. These studies have revealed the potential mechanisms of TCM in regulating the GM, including achieving its therapeutic effects by inhibiting the growth of harmful microorganisms, promoting the reproduction of beneficial microorganisms, and through the interaction of metabolites with host cells (Gong et al., 2020). In addition, the GM not only affects the efficacy of TCM, but also participates in the metabolism of TCM ingredients, affecting their bioavailability and efficacy. Research on TCM is not limited to its regulation of the GM, but also extends to the regulation of the microbiota-gut-organ axis. Current research shows that the GM interacts with multiple organs (such as liver, heart, and brain) through intestinal barrier, neuroendocrine, and immune mechanisms to form a complex regulatory network (Zhao et al., 2021). This regulatory mechanism of the microbiome-gut-X axis provides a new biological basis for the clinical application of TCM and may open up new directions for the integrated application of TCM in modern medicine.

The objective of this review is to examine the interaction between TCM and the GM, with a particular emphasis on its therapeutic potential in regulating the microbiome-gut-X axis. This review will examine the existing literature on the subject and analyse how TCM can improve health status by regulating the GM, with a particular focus on the prevention and treatment of common diseases. In addition, it will discuss future research directions, including the potential of personalized TCM treatments and the use of modern biotechnology to further explore the complex relationship between TCM and the GM.

## 2 Methodology and literature search strategy

A comprehensive literature search was conducted across a range of databases, including Elsevier SD, Web of Science, PubMed, and Google Scholar. The search strategy employed a combination of keywords, including “gut microbiota,” “traditional Chinese medicine,” “microbiota-gut-organ,” “gut-brain axis,” “gut-liver axis,” “gut-lung axis,” and “action mechanism.” The results included publications from 2000 to 2024, with a particular focus on recent papers. The objective was to collate data on the correlation between disparate distant organ systems and the GM, as well as the interaction between TCM and GM for systemic health and disease.

## 3 Mechanisms of gut microbiota influence on distant organ systems

**FIGURE 1:**
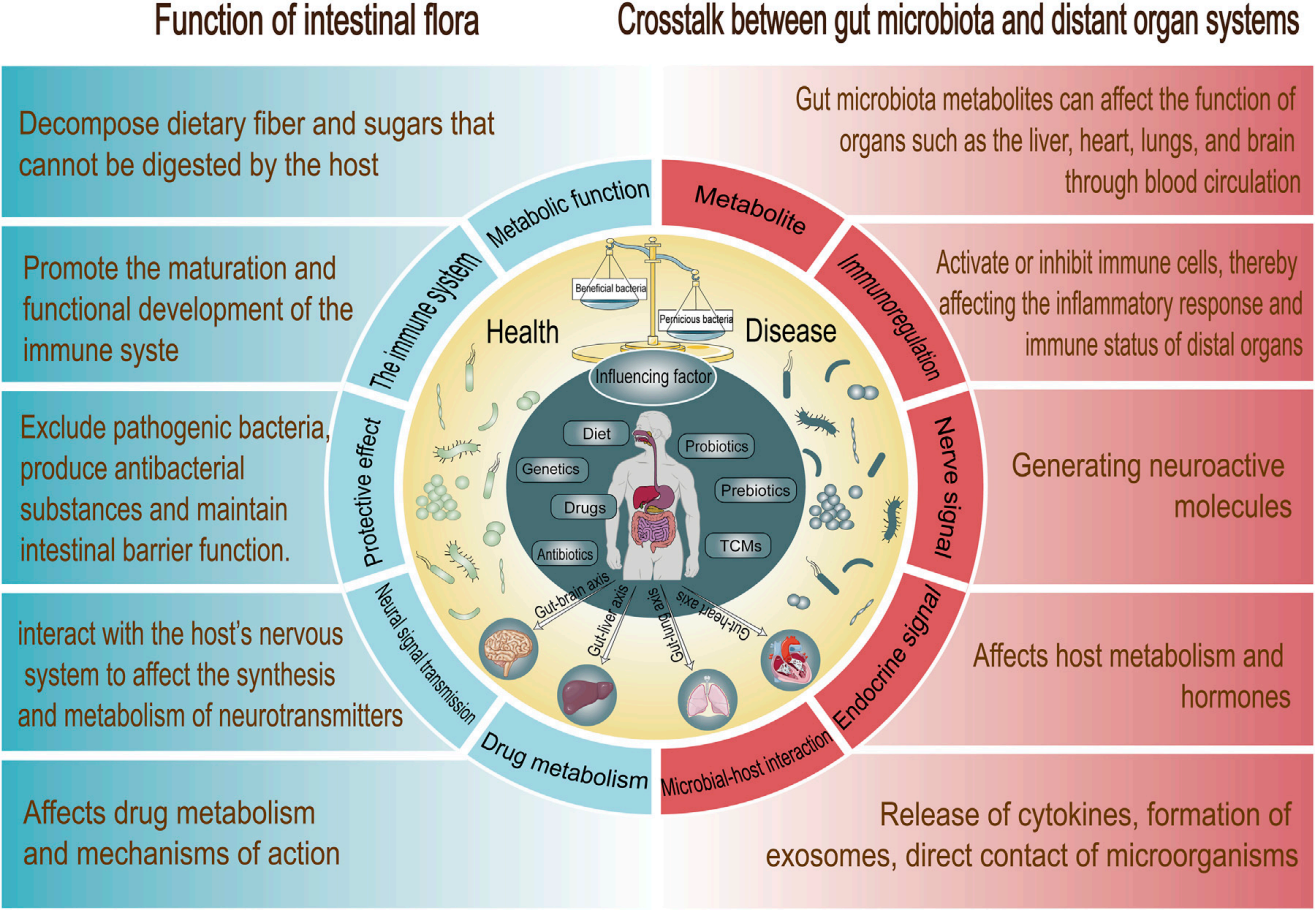
Gut microbiota function and microbiota-gut-X axis signaling communication pathways.

In recent years, the investigation of GM has emerged as a significant topic within the biomedical field. GM are not merely components of the digestive system; they are also believed to play a crucial role in systemic health and disease. An increasing body of evidence indicates that GM interact with distant organs via various pathways, thereby influencing their function and overall health (Figure 1).

### 3.1 The gut microbiota: a key player in health

The composition of the GM is highly intricate, typically comprising around 100 trillion microorganisms from over 1,000 distinct species. Research indicates that the GM of healthy adults is primarily composed of the following bacterial phyla (Hollister et al., 2014; Vijay and Valdes, 2022): *Firmicutes*, a group of bacteria that dominates the healthy human gut, is closely associated with energy extraction and fat metabolism. *Bacteroidetes*, on the other hand, are responsible for breaking down complex carbohydrates and promoting nutrient absorption. Additionally, other bacterial phyla, such as *Actinobacteria* and *Proteobacteria*, also play vital roles in maintaining intestinal health and function. The composition of the microbiome is influenced by various factors, including genetics, diet, geographical location, age, and medical treatments, which collectively determine the diversity and functionality of individual gut microbes (Panda et al., 2014; Ruan et al., 2020).

The main functions of the GM can be summarized into the following points: 1) Metabolic function (Fan and Pedersen, 2021): GM can decompose dietary fiber and sugars that cannot be digested by the host and convert them into short-chain fatty acids (SCFAs) to provide energy for the host. These SCFAs not only provide energy for intestinal cells, but also have immune-modulating and anti-inflammatory effects. 2) Development and regulation of the immune system (Dupont et al., 2020): The GM is involved in the development and regulation of the host immune system, helping to recognize and defend against pathogens. By interacting with intestinal epithelial cells, microorganisms can influence the activity of immune cells and promote the production of antibacterial proteins, thereby enhancing the host’s resistance to infection. 3) Protective effect (Chen et al., 2024): The microbiome plays a role in protecting the host by competitively excluding pathogenic bacteria, producing antibacterial substances and maintaining intestinal barrier function. This mechanism helps prevent the invasion of pathogenic microorganisms and reduces the risk of intestinal infection. 4) Neural signal transmission (Altamirano et al., 2020): GM interact with the host’s nervous system to affect the synthesis and metabolism of neurotransmitters, thereby affecting mood and behavior. The concept of gut-brain axis has attracted widespread attention, suggesting that the microbiome may play an important role in mental health. 5) Drug metabolism (Fan and Pedersen, 2021): The microbiome also affects drug metabolism and mechanisms of action. For example, certain drugs are metabolized by microbes in the gut, changing their bioavailability and side effects. This discovery provides new perspectives for personalized medicine.

The GM is one of the key factors in maintaining human health. Its complex composition and diverse functions jointly affect the host’s metabolism, immunity and overall health. Although the GM varies greatly among individuals, its functions are relatively conserved. This means that different individuals’ microbiomes may contain different combinations of species, but they may have similar functions such as metabolism and immune regulation (Rinninella et al., 2019). However, the composition of the microbiome is not static and may change due to factors such as diet, environment, drug use, etc., leading to microbial dysbiosis, which is closely related to the occurrence of various diseases, such as inflammatory bowel disease, obesity, diabetes, etc (Hills et al., 2019; Zhang, 2022).

### 3.2 Crosstalk between gut microbiota and distant organ systems: mechanisms and implications

Dysbiosis of the GM is associated with disease in a variety of distal organs. For example, studies have shown that changes in the GM may be linked to lung diseases (such as asthma) and joint diseases (such as rheumatoid arthritis), which are known as the “gut-lung axis” and “gut-joint axis.” These interactions suggest that the GM not only affects intestinal health but may also influence the function of other organs through complex signaling pathways (Chu et al., 2023). The communication mechanism between the intestinal tract and its flora and various organs, namely, the “gut-organ axis,” has become increasingly important in maintaining the health of various organs. Research has gradually revealed the complex mechanisms of action of the GM on affecting distal organ systems (Table 1). GM can affect distal organ functions in a direct way via the highly complex gut-organ axis by means of:

• Metabolite: Gut microbes can metabolize food components to produce SCFAs, amino acids, and other metabolites, which can affect the function of organs such as the liver, heart, lungs, and brain through blood circulation. For example, SCFAs can modulate immune responses and influence systemic inflammatory status (Shastry and Rekha, 2021; Marroncini et al., 2024).
• The immune system: GM can affect the immune response of the whole body by interacting with the host immune system. Microbial metabolites and signaling molecules can activate or inhibit immune cells, thereby affecting the inflammatory response and immune status of distal organs (Dong and Mayer, 2024).
• The neural network: Such as vagus nerve by producing bacterial metabolites, the intrinsic branches of the enteric nervous system, and the extrinsic parasympathetic and sympathetic branches of the autonomic nervous system (Sherwin et al., 2018).
• The endocrine system (via HPA axis): For example, the intestine can secrete peptide hormones (such as incretin and gastrin). These hormones play an important role in regulating appetite and metabolism, and their changes may affect the function of the liver and pancreas (Fava, 2014).

**TABLE 1:** Major studies related to the Microbiota-Gut-X Axis.

Research axis	Research topic	Method	Main findings	Ref.
Microbiota-Gut-brain Axis	The impact of the microbiome on mood and behavior	Using a germ-free mouse model to observe the impact of microbiota loss on emotional behavior	Microbiota-deficient mice exhibit anxiety- and depression-like behaviors, suggesting the importance of the microbiota in mood regulation	Osadchiy et al. (2019), Mayer et al. (2022)
	The relationship between microbiota and neuroinflammation	Assessing gut microbiome changes in neuroinflammatory markers in mouse models	Compositional changes in the microbiota are associated with neuroinflammation and may influence the development of neurodegenerative diseases	Singh et al. (2024)
	The mechanism of gut-brain signaling pathway	Analyze the expression of different signaling molecules (e.g., intestinal hormones, neurotransmitters) in mice	Gut hormones and neurotransmitters play key role in signaling between microbiota and central nervous system	Ding et al. (2020), Chakrabarti et al. (2022)
Microbiota-Gut-liver Axis	The relationship between microbiota and liver function	Studying the impact of altered microbiota on liver metabolic markers	Gut microbiota composition affects hepatic lipid metabolism and insulin sensitivity, suggesting that gut health is closely linked to liver health	Anand and Mande (2022)
	The role of gut microbiota in liver disease	Using animal models to assess the impact of gut microbiota on the progression of liver fibrosis	Dysbiosis of the gut microbiota is associated with the development of liver fibrosis, suggesting its potential in the treatment of liver disease	Anand and Mande (2022)
Microbiota-Gut-lung Axis	The relationship between the microbiome and respiratory health	Assessing the impact of microbiota on lung inflammation in mouse models	Alterations in the gut microbiota affect lung immune responses and may be associated with the development of asthma and other respiratory diseases	Shi et al. (2023)
	Impact of gut microbiota on pulmonary infections	Studying the role of the microbiota in a mouse model of lung infection	Gut microbiota affects severity of lung infection by modulating systemic immune response	Ribeiro et al. (2022)

The interaction between the GM and remote organ systems is a complex and dynamic process involving multiple mechanisms such as the production of metabolites, immune regulation, and neural signaling. These interactions not only affect the health of the gut but also have profound effects on physiological functions and disease states throughout the body.

#### 3.2.1 Research evidence on a pathogenic link between gut microbiota and neuropsychiatric disorders

Much evidence strongly indicates that there is a positive correlation between GM dysbiosis and the onset or expression of various neuropsychiatric disorders. These conditions encompass a range of neuropsychiatric conditions, including Alzheimer’s disease, anxiety, depression and Parkinson’s disease (Stevens et al., 2021). Such findings highlight the importance of gut health in the context of mental wellbeing and raise questions about potential therapeutic interventions targeting the GM for individuals suffering from these disorders. In addition to clinical observations, high-throughput genetic sequencing and metabolomics studies have reported notable alterations in the composition of gastrointestinal microbiota, as well as changes in fecal metabolic profiles that are associated with depressive disorders (Yu et al., 2017; Stevens et al., 2021). These scientific advancements provide deeper insights into the relationship between GM and neuropsychiatric conditions.

• Alzheimer’s disease (AD): A large number of clinical studies and animal experiments have revealed the correlation between GM and AD. For example, one study analyzed the composition of the GM in people with AD and found that the abundance of specific microbes was associated with cognitive decline (Cammann et al., 2023; Zhang et al., 2024a). Additionally, laboratory studies show that changes in the GM can affect behavior and cognition in mice modeled with AD, providing further evidence to support this hypothesis (Chen et al., 2022; Dissanayaka et al., 2024). In microbial transfer experiments, researchers transplanted the GM of AD patients into experimental animals (such as rats) and observed that these animals showed neurogenesis (i.e., the generation of new neurons) and decreased dendritic formation. This finding suggests that the GM of people with AD may further worsen the condition by interfering with the growth and connections of neurons (Grabrucker et al., 2023). Although studies have shown that there is a link between GM and AD, the specific mechanism remains unclear. There are currently three main mechanisms: 1) Amyloid production (Cheng et al., 2021): Some studies have proposed that GM can produce amyloid proteins, which are an important pathological feature of AD. These proteins can enter the blood circulation and cross the blood-brain barrier, and then aggregate and form in the brain. 2) Inflammatory response (Cammann et al., 2023; Liang et al., 2024): The imbalance of GM may lead to the impairment of intestinal barrier function and increase intestinal permeability, thereby allowing bacterial components and metabolites to enter the blood. These substances may trigger a systemic inflammatory response, thereby affecting the nervous system and promoting the progression of AD. 3) Impact of metabolites (Wu Y. et al., 2022): Metabolites produced by GM, such as SCFAs, have an important impact on the survival and function of neurons. SCFAs not only participate in energy metabolism, but also regulate the synthesis of neurotransmitters and affect cognitive function.
• Anxiety and depression: Studies have found that the GM of patients with depression exhibits significant dysbiosis, which is significantly different from healthy individuals. For example, the number of certain beneficial bacteria (such as *Lactobacillus* and *Bifidobacterium*) is reduced in patients with depression, while the number of harmful bacteria is increased (Butler et al., 2023; Yuan et al., 2024). This microbial dysbiosis may lead to changes in metabolites that affect neurotransmitter synthesis and function, negatively impacting mood and behavior. In addition, the GM can influence brain function through the production and regulation of various neurotransmitters. For example, GM can synthesize serotonin, a neurotransmitter closely related to mood regulation. Studies have shown that the microbial community in the intestines can affect serotonin levels through different pathways, thereby affecting the occurrence of anxiety and depressive symptoms (Kumar et al., 2023; Yang R. et al., 2023).
• Parkinson’s disease (PD): Clinical studies show that about 30% of PD patients often show some intestinal symptoms, such as constipation and difficulty swallowing, before the onset of motor symptoms. These symptoms may be early warning signs of the disease, suggesting that GM and their imbalance play an important role in the early development of PD (Zhu et al., 2022; Zhang M. et al., 2023). Some clinical trials have found that the GM of patients with PD shows specific changes, such as a decrease in the number of certain beneficial bacteria and an increase in the number of pathogenic bacteria. This imbalance may lead to intestinal inflammation and other metabolic problems (Zhu et al., 2022). Some studies have suggested that intestinal inflammation caused by GM may be a key factor leading to α-synuclein aggregation, and this aggregation is considered to be one of the pathological characteristics of PD (Moustafa et al., 2021; Chan et al., 2022; Warnecke et al., 2022). The inflammatory state of the intestine may promote the accumulation of harmful proteins, thereby affecting the normal function of the nervous system. In mouse experiments, a decrease in locomotor capacity and an increase in α-synuclein were observed after transplantation of GM from PD patients, further supporting the critical role of GM in the development of PD (Li B. et al., 2023).

#### 3.2.2 The influence of gut microbiota composition on the development and progression of liver diseases

Physiologically, there is a two-way communication between the intestine and the liver through the biliary system and portal vein, which allows metabolites and microorganisms in the intestine to quickly affect liver function. Metabolites of intestinal flora, such as SCFAs and other microbial metabolites, can be absorbed by the liver through enterohepatic circulation, thereby regulating liver metabolism and immune responses (Jiang and Schnabl, 2020; Li B. et al., 2022). Dysbiosis of the GM is considered an important factor in the development of liver disease. Studies have shown that damage to the intestinal barrier function (i.e., the “leaky gut” phenomenon) can lead to the transfer of bacteria and their metabolites to the liver, thereby triggering liver inflammation and damage (Konturek et al., 2018). This bacterial transfer is not only the pathological basis of liver disease, but may also promote the aggravation of liver inflammation and the progression of the disease. Furthermore, metabolites produced by GM play dual roles in liver disease. On the one hand, some metabolites (such as SCFAs) help maintain the health of the liver and have anti-inflammatory and protective effects. On the other hand, certain pathogenic bacteria and the toxins they produce may cause damage to hepatocytes and chronic inflammation of the liver (Schnabl and Brenner, 2014). Therefore, the composition and metabolic activity of GM are crucial in the occurrence and development of liver diseases.

Some studies have suggested that GM may affect the occurrence and development of liver diseases through the following three mechanisms: 1) Inflammatory response (Schwenger et al., 2019): Metabolites (such as endotoxins) produced by GM can trigger an inflammatory response in the liver, leading to liver cell damage and fibrosis. 2) Metabolic regulation (Kanmani et al., 2020): GM affect the function of the liver by affecting the metabolic processes of the host, including lipid metabolism and glucose metabolism. 3) Immunomodulation (Wang R. et al., 2021; Wang H. et al., 2022): Changes in the microbiome can affect the distribution and activity of immune cells in the liver, thereby affecting the immune response of the liver. Furthermore, bile salts represent the primary organic solutes of bile and are synthesized by the liver. The majority of bile salts are primary bile salts, which play a crucial role in the digestive process. The available evidence indicates that there is a bidirectional relationship between GM and bile salts (Li et al., 2023b). On the one hand, the GM convert these primary bile salts into secondary bile salts through a biotransformation process, thereby increasing the diversity of bile salts (Guo et al., 2022). The metabolic activity of GM not only affects the composition of bile salts, but also has a significant impact on the size and concentration of bile salt pools. In turn, bile salts act in the intestines to inhibit bacterial overgrowth and protect the intestines from inflammation, thereby maintaining body health (Oscar et al., 2017).

• Acute liver injury: Acute liver injury is sudden damage to the liver caused by a variety of reasons. Studies have shown that when the intestinal mucosal barrier is damaged, intestinal bacteria and their products will migrate to the liver, causing a series of immune damage and inflammatory reactions, and ultimately causing liver damage (Zhang et al., 2024b). This phenomenon emphasizes the importance of the interaction between the gut and the liver, the “gut-liver axis,” in acute liver injury. In experiments on mice, the researchers used antibiotics to clear their GM and found that the mice showed more severe symptoms of liver damage after they were treated with hepatotoxins, such as carbon tetrachloride or hepatitis virus. This suggests that a loss of GM may render the liver more susceptible to damage (Chen et al., 2021; Hu et al., 2024). Other studies have pointed out that specific intestinal bacterial communities may play a protective role in the occurrence of liver injury (Wang K. et al., 2022). In addition, the GM also plays an important role in drug-induced liver injury. Studies have found that certain drugs may induce varying degrees of liver toxicity under the regulation of GM. Changes in the composition of GM are closely related to drug metabolism and toxic reactions, which provides a new perspective for understanding the effects of drugs on the liver (Huang et al., 2024).
• Viral Hepatitis: Many clinical and experimental studies have supported the role of GM in the development of liver disease. For example, one study found that changes in the GM were associated with disease progression in patients with chronic hepatitis B, and the characteristics of the GM could serve as potential biomarkers for monitoring disease progression (Sehgal et al., 2020; Khabarova and Ya, 2024). In addition, experimental studies have shown that transplantation of GM (such as fecal microbiota transplantation) may become an emerging strategy for the treatment of viral hepatitis and can improve patients’ clinical outcomes (Yang et al., 2020). According to the theory of the gut-liver axis, GM affects the development of viral hepatitis mainly by regulating the inflammatory response of the liver and intestines. Studies have found that GM can promote the healthy function of the liver by producing SCFAs, vitamin K, vitamin B12 and other nutrients. In addition, GM can also regulate the defense against viral infection by affecting the response of the host immune system (Lu et al., 2021; Lv et al., 2021).
• Non-alcoholic fatty liver disease (NAFLD): Research shows that imbalance of GM may play an important role in the pathogenesis of NAFLD. The onset of NAFLD is closely related to metabolic factors such as diet, obesity, and insulin resistance, and these factors are in turn affected by GM. GM can affect the health of the liver by regulating the host’s metabolic state, inflammatory response, and intestinal barrier function (Yu et al., 2016). Multiple studies have revealed that the composition of GM in NAFLD patients is significantly different from that in healthy individuals. Specifically, the number of some beneficial bacteria in the GM of patients with NAFLD is reduced, while some potentially harmful bacteria (such as some bacteria from the phyla *Firmicutes* and *Proteobacteria*) are relatively increased. These changes may lead to the weakening of intestinal barrier function, which in turn triggers the penetration of intestinal contents into the blood circulation and promotes liver inflammation and fat deposition (Cai et al., 2024; Zeng et al., 2024). In addition, studies have found that intestinal microbial metabolites SCFAs can improve the pathological process of NAFLD by inhibiting liver fat synthesis and enhancing insulin sensitivity. However, in NAFLD patients, the production of SCFAs is often inhibited, further aggravating the progression of the disease (Pan et al., 2020; Jadhav et al., 2024).

#### 3.2.3 The role of gut microbiota in driving lung disease progression: experimental evidence and clinical reality

The gut-lung axis refers to the two-way communication channel between the GM and the lung. Although the two organs are anatomically separate, studies have shown that complex biochemical signaling mechanisms exist between them. GM may influence lung health through their metabolites, cell signaling, and modulation of immune responses (Enaud et al., 2020). In some common lung diseases, such as asthma, chronic obstructive pulmonary disease, and pulmonary fibrosis, dysbiosis of the GM is considered an important factor in the pathological process. Studies have found that there are significant differences in the GM of asthma patients compared with healthy people. These differences are related to the patient’s inflammation level and disease severity (O’dwyer et al., 2019; Zhang D. et al., 2020). In mouse models, researchers found a significant correlation between changes in the GM and the progression of lung disease. By changing the mice’s diet or using probiotics, the researchers were able to modulate the composition of the GM and observe improvements in lung inflammation and lung function. These results indicate that the GM not only plays a key role in the occurrence of lung diseases, but may also become a potential therapeutic target (O’dwyer et al., 2019; Wang and Wang, 2024).

• Chronic obstructive pulmonary disease (COPD): Multiple clinical studies have shown that changes in GM are closely related to the severity and clinical manifestations of COPD. For example, a study of 73 healthy controls and 67 COPD patients found that COPD patients had significantly lower levels of SCFAs in stool samples than healthy people. SCFAs such as acetic acid, propionic acid, and butyric acid are essential for maintaining intestinal health and immune regulation, which may affect the course of COPD (Kirschner et al., 2021; Li et al., 2021; Lai et al., 2024). In addition, COPD patients are often accompanied by digestive system symptoms, such as bloating, constipation, and diarrhea, and these symptoms may be related to the imbalance of GM (De Nuccio et al., 2022). Studies have also found that COPD patients who received bacterial transplants experienced improvements in lung function and quality of life, further supporting the role of GM in COPD (Lim et al., 2023).
• Asthma: Studies have found that the composition of GM is closely related to the occurrence of asthma. Specifically, dysbiosis of the GM can lead to an abnormal response of the immune system, thereby increasing the risk of asthma. Many studies have shown that changes in early GM are significantly associated with the occurrence of asthma in children. For example, studies have shown an association between microbial composition at 1 year of age and asthma diagnosis at 5 years of age, suggesting that early microbial exposure is critical for the development of the immune system (Stokholm et al., 2018). Studies have shown that the use of antibiotics can change the composition of GM and enhance the response to allergens in mice, which further supports the connection between GM and asthma (Stiemsma and Turvey, 2017). Some studies have pointed out that GM imbalance may lead to dysfunction of T regulatory cells (Tregs), which is closely related to the pathogenesis of asthma (Zheng et al., 2023). In addition, the health status of GM is also related to the occurrence of allergic diseases, suggesting that GM may participate in the occurrence of asthma by affecting the systemic immune response (Kang et al., 2017).
• Pulmonary fibrosis: Some studies have investigated the association between GM and pulmonary fibrosis through cross-sectional studies, and the results show that the composition of the GM in patients with pulmonary fibrosis is significantly different from that in healthy controls. These findings indicate that specific GM may be closely related to the occurrence of pulmonary fibrosis, and suggest that changes in GM may be a biomarker of pulmonary fibrosis (Puiu et al., 2024). Studies of improving pulmonary fibrosis by modulating GM (such as using probiotics or prebiotics) have also shown positive effects in animal experiments. For example, the application of certain probiotics can reduce the levels of inflammatory factors in lung tissue and reduce the degree of pulmonary fibrosis. These results suggest that intervention of GM may slow or reverse the process of pulmonary fibrosis to a certain extent (Costa et al., 2022; Guo and Yang, 2024). Studies have found that changes in GM may participate in the pathological process of pulmonary fibrosis by affecting the host’s immune response, inflammatory response and the expression of related cytokines. For example, certain metabolites produced by GM may affect immune cells in the lungs through blood circulation, thereby changing the inflammatory environment in the lungs. This provides mechanistic support for understanding the relationship between intestinal flora and pulmonary fibrosis (Puiu et al., 2024).

In summary, the GM is of great significance in maintaining health and promoting disease, and its mechanisms of action are still being revealed. Future research will further explore its relationship with remote organ diseases and explore its potential therapeutic targets and strategies. As technology advances and research deepens, the GM may become an important area for understanding and treating a variety of diseases. Not only will this help reveal the complexity of the microbiome, it may also provide us with new health interventions to address various diseases associated with microbiome dysbiosis. For example, by adjusting diet, using probiotics or synthetic microbiota, it may be possible to effectively improve the health status of the host. In addition, individual differences in GM also suggest that we need to consider personalized medicine strategies during the treatment process.

### 3.3 Gut microbiota manipulation for future health interventions

The treatment of GM shows great promise for the future in addressing a number of intractable diseases. The association between GM dysbiosis and neurological, hepatic, and pulmonary disorders has been proven, and interventions targeting gut flora, such as probiotics, prebiotics, and fecal microbial transplantation (FMT), are emerging as a promising therapeutic strategy, especially when conventional pharmacological treatments have limited efficacy.

#### 3.3.1 Probiotics and prebiotics

Probiotics are defined as live microorganisms that confer benefits to the host, with the most commonly studied examples being *lactobacilli* and *bifidobacteria*. A growing body of evidence suggests that probiotics can alleviate the symptoms of intestinal disorders by modulating the GM, enhancing intestinal barrier function, and modulating the immune response (Skoufou et al., 2024). For example, clinical studies on inflammatory bowel disease and irritable bowel syndrome have demonstrated that probiotics are efficacious in reducing symptoms and improving quality of life (Ji et al., 2023). Furthermore, the use of probiotics has shown promise in terms of enhanced drug metabolism and efficacy (Purdel et al., 2023).

While Prebiotics are substances that selectively promote the growth and activity of probiotics within the gut. Commonly, oligosaccharides and cellulose are considered prebiotics. The mechanism of action of prebiotics is primarily through the provision of nutrients that facilitate the colonization of beneficial flora, thereby improving the composition and function of the GM (Ji et al., 2023). Studies indicate that the consumption of prebiotics can positively influence the composition and function of the GM, which in turn can have a beneficial impact on host health, particularly in the context of chronic disease management (Al-Fakhrany and Elekhnawy, 2024).

#### 3.3.2 Fecal microbiota transplantation

As an emerging therapeutic approach, fecal microbiota transplantation (FMT) has demonstrated efficacy in controlling antibiotic-associated diarrhea and *Clostridium difficile* infections. By introducing microbiota from the feces of healthy donors into the patient’s gut, the approach aims to restore the balance of the patient’s GM and thereby improve clinical outcomes. FMT was initially used primarily to treat conditions associated with intestinal dysbiosis, such as recurrent *C. difficile* infection, but its potential range of applications is expanding, particularly in distal organs. Shows promise in the treatment of the disease.

• Chronic kidney disease: Studies have shown that FMT can improve the microbial community structure of kidney transplant recipients and reduce the colonization rate of multi-drug-resistant bacteria. This finding provides a theoretical basis for the application of FMT in patients with chronic kidney disease, especially in patients who need to maintain renal function (Woodworth et al., 2023).
• Liver disease: Patients with liver disease are often accompanied by intestinal flora imbalance. FMT is expected to reduce liver inflammation and fibrosis by improving the composition of intestinal microbiota. For example, some studies suggest that FMT can improve gut health in people with cirrhosis, which in turn affects liver function (Goldenberg et al., 2023).
• Cardiovascular disease: Increasing evidence shows that the GM is closely related to cardiovascular health. FMT may have a positive impact on patients with cardiovascular disease by changing the composition of the GM and reducing the inflammatory response. Some preliminary studies suggest that FMT may help improve metabolic status and cardiac function in patients with heart disease (Hao et al., 2023).

Although FMT has great application potential, more research is needed to determine its safety and effectiveness. Existing data suggest that FMT is safe in many cases and has a particularly high success rate in the treatment of recurrent *C. difficile* infection (Lin et al., 2018). However, the efficacy and safety of FMT in immunocompromised patients, such as organ transplant recipients, remain unclear, and further clinical trials are needed to evaluate its potential risks and benefits (Thanush and Venkatesh, 2023).

There are significant distinctions between GM therapy and conventional pharmaceuticals, particularly with regard to the following factors: 1) Mechanism of action: Traditional pharmaceuticals typically exert their effects by directly targeting pathogens or by interfering with biochemical pathways. In contrast, GM therapy influences the health of the host by modulating the microbiome. For example, GM can produce a variety of chemicals that can interfere with the metabolism of drugs or enhance their effects, which provides us with different therapeutic ideas (Rahman et al., 2022). 2) Side effects: Conventional pharmaceuticals frequently elicit adverse effects, including gastrointestinal distress, liver and kidney injury, and the exacerbation of underlying ailments. In contrast, GM therapy tends to have a lower incidence of adverse effects, as it relies on the use of natural microorganisms to promote health, rather than introducing foreign chemicals. This renders GM therapy a potentially safer option in certain cases (Zhao et al., 2023). 3) Wide range of therapies: GM therapies can be utilized to target a multitude of conditions, including some complex diseases such as autoimmune disorders and allergic diseases, thereby demonstrating the potential for a wider range of applications. While traditional medications are typically designed to target specific conditions, GM therapies may affect multiple physiological systems simultaneously, resulting in systemic health benefits (Rahman et al., 2022).

However, it is worth noting that the efficacy of GM therapy is not solely contingent on the specific type and quantity of microorganisms transplanted, but is also influenced by individual variations in the host. This suggests that individualized treatment regimens may become a crucial aspect in future clinical applications. For example, it has been demonstrated that different individuals exhibit disparate responses to the same FMT treatment, which may be attributable to factors such as an individual’s genome, diet, and lifestyle (Yang Y. et al., 2023; Sahle et al., 2024). Consequently, future studies must prioritize the characterization of an individual’s microbiome and its impact on treatment response.

## 4 Integrating traditional Chinese medicines with gut microbiota for microbiota-gut-organ axis regulation

In recent years, with the increasing public attention to intestinal health, GM have become a hot topic in TCM research. The GM and intestinal barrier are considered to be one of the important targets for TCMs to function and treat diseases (Che et al., 2022). Progress in this research field provides a new perspective for our understanding of the role of TCMs in the regulation of the microbiome-gut-organ axis.

### 4.1 Regulation mechanism of traditional Chinese medicines on gut microbiota

TCM is an ancient medical system with a history of thousands of years. Its theoretical basis includes the theory of yin and yang five elements theory, qi and blood theory, viscera theory, etc. These theories form the basis for TCM diagnosis and treatment of diseases. In recent years, with the deepening of research on the GM, the potential of TCM in regulating GM has been gradually recognized and valued (Table 2) (Zhang R. et al., 2020).

**TABLE 2:** Overview of major bacterial groups and functions in gut microbiome and TCM effects.

Bacterial group	Relative abundance (%)	Key functions	Effects of TCM	Ref.
*Firmicutes*	60–80	Fermentation of dietary fibers Production of (SCFAs)	TCM can help restore balance by increasing beneficial *Firmicutes* in dysbiosis	Ribeiro et al. (2022)
*Bacteroidetes*	15–30	Breakdown of complex carbohydrates Maintenance of gut barrier integrity	TCM may modulate *Bacteroidetes* to enhance metabolic health	Zhang et al. (2020a)
*Proteobacteria*	1–10	Involved in inflammation and immune responses	Increased levels may indicate dysbiosis TCM aims to reduce harmful *Proteobacteria*	Li et al. (2022c)
*Actinobacteria*	1–5	Production of vitamins Protection against pathogens	TCM can support the growth of beneficial *Actinobacteria*	Rinninella et al. (2019)
*Fusobacteria*	<1	Role in oral and gut health Involvement in inflammatory conditions	TCM may help in managing conditions associated with *Fusobacteria*	Rowland et al. (2018)
*Verrucomicrobia*	<1	Mucus degradation Regulation of inflammation	TCM may enhance the abundance of *Verrucomicrobia* to improve gut health	Lin et al. (2021)
*Desulfobacterota*	<1	Sulfate reduction Impact on gut health	TCM interventions may aim to balance their levels for better gut function	Li et al. (2022c)

**FIGURE 2:**
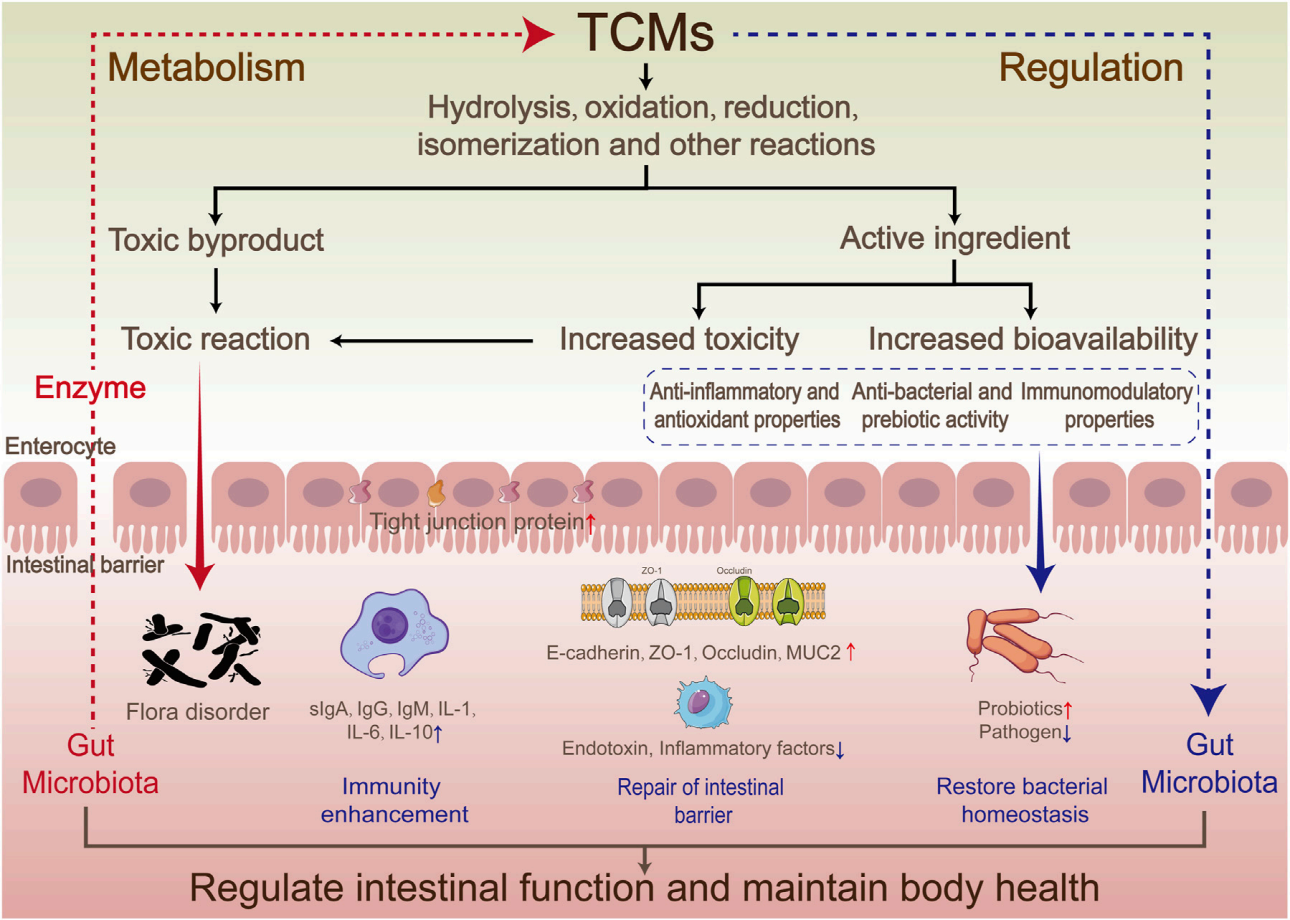
Interaction mechanism between traditional Chinese medicines and gut microbiota.

Numerous studies have demonstrated that TCMs can regulate GM through multiple mechanisms, including restoring the balance of flora, affecting metabolic function, activating immune function, repairing the intestinal barrier, regulating flora metabolites, inhibiting the growth of harmful bacteria, and promoting microbial diversity (Figure 2). These mechanisms work together to help maintain intestinal health and prevent and treat a variety of diseases (Wu Z. et al., 2022).

#### 4.1.1 Regulating the ratio of probiotics to pathogenic bacteria

It is vital to maintain a certain ratio between probiotics and pathogenic bacteria as it can ensure the stability of the gut microecosystem. An imbalance and pathogenicity can occur when there is an excess or deficiency of one or more bacterial strains. Studies have shown that certain TCMs exhibit antimicrobial and prebiotic activities, which can restore the balance between probiotics and pathogenic bacteria in the intestinal tract. In a study, the effects of salidroside on the abundance and composition of GM in mice with ulcerative colitis (UC) were examined. The study found that after oral administration of salidroside, the abundance of enteric pathogenic bacteria such as *Turicibacter*, *Alistipes*, and *Romboutsia* significantly decreased. Conversely, the abundance of the beneficial bacterium *Lactobacillus* increased significantly, resulting in a significant therapeutic effect on the UC mice. However, the therapeutic effect of salidroside disappeared when antibiotics were used to deplete GM. It turns out that salidroside can exert anti-inflammatory effects by increasing the number of intestinal probiotics and inhibiting the number of pathogenic bacteria (Liu et al., 2023).

Most TCMs contain polysaccharides as their main active ingredients. As they are not easily digested, polysaccharides do not have a direct effect on the host. However, they can improve the intestinal environment and serve as a substrate for probiotics, promoting their growth and addressing GM dysbiosis through a prebiotic effect [41]. An example is the polysaccharides found in ginseng (*Panax ginseng* C.A.Mey.) in Du-Shen-Tang, which cannot be directly absorbed and utilized by the intestinal tract. However, they can promote the metabolism of ginsenoside elements such as ginsenosides Re and Rc in the intestinal tract. Additionally, they can promote the growth of probiotic bacteria such as *Lactobacillus* spp. and *Bacteroides spp.* (Zhou et al., 2016).

Furthermore, TCMs can indirectly influence GM metabolism by regulating the ratio of intestinal probiotics to pathogenic bacteria. Studies have found that bacterial metabolites, such as SCFAs, can contribute to the treatment of diseases by improving metabolic disorders, reducing inflammation, and repairing the intestinal barrier. For example, Bletilla striata (*Bletilla striata* (Thunb.) Rchb.f.) oligosaccharides improve high-fat diet-induced metabolic syndrome by reversing GM dysbiosis and restoring homeostasis of gut metabolites such as bile acids, SCFAs, and tryptophan catabolite metabolites (Hu et al., 2020). Rhein, derived from the Chinese herb rhubarb (*Rheum palmatum* L.), has the ability to alter the composition of the GM. As a result of this modification, there is an increase in the abundance of *Lactobacilli*, which indirectly affects purine metabolism in the intestinal tract and reduces the production of uric acid in the intestinal epithelium. This relieves UC symptoms, resulting in their alleviation (Wu et al., 2020).

However, some TCMs can disrupt the structure of GM. For example, the combination of Qianjinzi (*Euphorbia lathyris* L.) and Gancao (*Licorice*) can increase the levels of *S247ukn*, *Candidatus arthromitus* and other endotoxin-synthesizing and intestinal immunity-related bacteria. This can lead to abnormalities in the structure of the GM macro genome. As a result, abnormalities can occur in the structure of the GM macro genome. Enhancing the expression of genes involved in aromatic amino acid breakdown and mucus breakdown may improve their functionality. This, in turn, may lead to an increase in the production of toxic substances, such as intestinal uremic toxins, which can elevate the risk of disease or exacerbate existing conditions (Wang L. et al., 2022).

#### 4.1.2 Protecting the intestinal mucosal barrier

The intestinal mucosal barrier is a system that comprises intestinal mucosal epithelial cells, chemicals present on the surface of the intestinal mucosa (such as HCO3-), intestinal microorganisms, and molecules of intestinal immune cells. Its function is to maintain the internal environment of the body and intestinal homeostasis, preventing the entry of bacteria, toxins, and other harmful substances into the intestinal tract. This is crucial in preventing the invasion of pathogenic bacteria and reducing inflammation. Multiple studies have demonstrated that TCMs, particularly those possessing anti-inflammatory and antioxidant properties, can safeguard the integrity of the intestinal mucosa through various mechanisms, such as regulating the structure of the GM, reducing endotoxin levels in the serum, and attenuating inflammatory factors.

Animal studies have shown that *Ganoderma lucidum* (Curtis) P. Karst. extract inhibits the growth of intestinal *Firmicutes* and *Bacteroidetes*, reduces the occurrence of endotoxemia, and protects the integrity of the intestinal mucosal barrier function in mice fed a high-fat diet (Chang et al., 2017). The combination of rhubarb and astragale (*Astragalus mongholicus* Bunge) has been shown to improve intestinal mucosal damage, reduce intestinal mucosal permeability, and inhibit endotoxin and GM translocation in rats with chronic renal failure (Yuping et al., 2017). Lin et al. discovered that Jianpi Yichang Powder exhibited a protective effect on the immune barrier in rats with UC. The mechanism was related to promoting the expression of heat shock protein-70 protein and mRNA in colon tissue (Lin et al., 2017).

Indigo and indirubin are isomeric active molecules found in the TCM indigo naturalis (*Indigofera* L.). They possess anti-inflammatory and immunomodulatory effects, regulate intestinal microbial composition, modulate oxidative stress, and promote intestinal mucosal repair. Xie et al. (2023) conducted a study to investigate the effects of co-administering indigo and indirubin in a colitis model. They found that all treatment groups exhibited improvements in disease symptoms, with the co-administration of indigo and indirubin being the most effective. In comparison to the groups that received indigo or indirubin alone, the co-administration group exhibited an upregulation in the expression of tight junction proteins, including E-cadherin, occludin, Zona Occludens 1, and Recombinant Mucin 2, in the colon. As a result, there was an improvement in intestinal permeability and a significant enhancement in intestinal barrier function. Further analysis revealed that the synergistic improvement in intestinal barrier function by indigo and indirubin is attributed to the integration of indirubin’s anti-inflammatory and GM-regulating abilities, as well as indigo’s immune and ROS/RNS regulation benefits.

#### 4.1.3 Enhancement of immune function

Immunomodulation is a crucial mechanism through which most TCMs exert their pharmacological effects. Relevant studies have shown that TCMs and their combinations have the ability to influence the body’s immune system by promoting the growth of intestinal probiotics and inhibiting the proliferation and colonization of potentially pathogenic bacteria, thereby achieving immune-boosting effects. Studies have shown that germ-free mice exhibit significantly weaker immune systems compared to normal mice. However, when they are colonized with normal gut microbes, their immune function is restored to normal levels (Ganal et al., 2012).

The intestinal bacterium *Bifidobacterium dentium* can stimulate the secretion of IL-1 and IL-6 from immune cells, thereby promoting the differentiation and maturation of B lymphocytes. Additionally, it enhances the killing function of NK cells and the proliferation of T lymphocytes (González Olmo et al., 2021). In an immunosuppressed mouse model, dandelion (*Perdicium capense* L.) and dangshen (*Codonopsis pilosula* Nannf.) significantly increased the thymus and spleen indices of immune organs, immunoglobulins (such as sIgA and IgG), and leukocyte counts of model mice. These effects may be attributed to the observed increase in abundance and diversity of *B. dentium* and *Lactobacillus* in the intestinal tract of the model mice (Wei et al., 2019). Sulforaphene extracted from semen raphani (*Raphanus raphanistrum* L.) has been shown to alleviate GM structural disorders and reverse colitis associated with an increase in intestinal T cells. Furthermore, Sulforaphene selectively inhibits the proliferation of pathogenic bacteria such as colorectal *Shigella* spp. and *Helicobacter pylori*, while promoting beneficial bacteria such as *Lactobacillus*. Intracellular components of *Lactobacillus* have been discovered to stimulate the growth of IL-17+γδ T cells. Increased IL-17A levels can then restore the compromised subcellular sites of closure proteins and safeguard the integrity of the colonic epithelial barrier (Li et al., 2017).

Furthermore, another study showed that the immune effects of TCMs can also be mediated by GM metabolites. Dendrobium officinale (*Dendrobium officinale* Kimura & Migo) polysaccharides have been shown to possess immunomodulatory activity. Through animal experiments, Li et al. discovered that these polysaccharides increased GM diversity in mice and promoted the production of more butyrate by the butyrate-producing strain *Parabacteroides_sp_HGS0025*. Additionally, the presence of *Parabacteroides_sp_HGS0025* was positively correlated with the production of butyrate, IgM, IL-10, and TNF-α in the intestine and blood of mice, respectively. This suggests that the immune effects of Dendrobium officinale polysaccharides may be mediated by butyrate, providing new evidence to support basic research on the effects of plant polysaccharides on immunity (Li D. S. et al., 2020).

The above-mentioned studies suggest that TCMs can modulate the GM through its unique ingredients and diverse mechanisms of action. However, there are certain differences and similarities in the composition and mechanism of action of TCMs. Some studies on commonly used formulas, Chinese medicine extracts, and Chinese medicine active ingredients to regulate intestinal flora are summarized in Table 3.

**TABLE 3:** Chinese herbal products regulate the gut microbiota composition.

Category	Chinese herbal products	Composition/Source	Disease model	Method	Changes in gut microbiota composition	Ref.
Formulas	Shenlian Decoction	*Coptis chinensis* Franch. [Ranunculaceae; Coptidis rhizoma] and *Panax ginseng* C.A.Mey. [Araliaceae; Ginseng radix]	Diabetes	16S rRNA	Prevotellaceae*↓*, Rikenellaceae*↓*, *and Helicobacteraceae↓*, and *Bacteroidaceae↑*	Sun et al. (2022)
	Dengzhan shengmai formula	*Erigeron breviscapus* (Vaniot) Hand.-Mazz. [Asteraceae; Erigeron], *Panax ginseng* C.A.Mey. [Araliaceae; Ginseng radix], *Schisandra chinensis* (Turcz.) Baill. [Schisandraceae; Schisandra], and *Ophiopogon japonicus* (Thunb.) Ker Gawl. [Asparagaceae; Ophiopogon]	Cerebral ischemia	Fecal microbiota transplantation and 16S rRNA	*Bacteroidetes↑* and *Firmicutes↓*	Guo et al. (2023)
	Qisheng Wan formula	*Poria cocos* (Schw.) Wolf [Polyporaceae; Poria], *Cinnamomum cassia* Presl [Lauraceae; Cinnamomum], *Polygala tenuifolia* Willd. [Polygalaceae; Polygala], *Panax ginseng* C. A. Mey. [Araliaceae; Panax], *Asparagus cochinchinensis* (Lour.) Merr. [Asparagaceae; Asparagus], *Acorus tatarinowii* Schott [Acoraceae; Acorus], and *Lycium chinense* Mill. [Solanaceae; Lycium]	Alzheimer’s disease	16S rDNA	*Epsilonbacteraeota↓*, *Tenericutes↓*, *Verrucomicrobia↑*, *Actinobacteria↓*, *Patescibacteria↓*, and *Deferribacteres↓*	Xiong et al. (2022)
Extracts	Honokiol	*Magnolia officinalis* Rehder & E.H.Wilson [Magnoliaceae; Magnolia]	Obesity	16S rRNA	*Akkermansia↑*, *Bacteroides↑* and *Oscillospira↓*	Ding et al. (2019)
	*Cichorium pumilum* Jacq Extract	*Cichorium pumilum* Jacq. [Asteraceae; Cichorium]	Hepatic fibrosis	16S rDNA	*Firmicutes/Bacteroidota↑* and *Ruminococcus↑*	Han et al. (2021)
	*Acanthopanax senticosus* Harms	*Acanthopanax senticosus* (Rupr. et Maxim.) Harms [Araliaceae; Acanthopanax]	Parkinson’s disease	Metagenome	*Firmicutes↑*, *Actinobacteria↓*, *Clostridium↑ and Akkermansia↓*	Lu et al. (2023)
Active ingredients	Forsythiaside A	Forsythia suspensa (Thunb.) Vahl [Oleaceae; Forsythia]	Liver fibrosis	16S rRNA	*Bacteroidetes↑* and *Firmicutes↓*	Fu et al. (2022)
	*Ramulus Mori (Sangzhi) a*lkaloids	*Ramulus Mori* [Moraceae; Morus]	Diabetic nephropathy	Fecal microbiota transplantation and 16S rRNA	*Dubosiella↑* and *Lactobacillus↑*	Liu et al. (2024)
	*Zanthoxylum bungeanum* amides	*Zanthoxylum bungeanum* Maxim. [Rutaceae; Zanthoxylum]	Nonalcoholic fatty liver	16S rRNA	*Firmicutes/Bacteroidota↓, Allobaculum↑, Bacteroides↑ and Dubosiella↑*	Peng et al. (2024)

First of all, Chinese medicine formula is usually composed of a variety of single Chinese medicines, which regulate GM through synergistic effects. For example, research shows that certain Chinese medicine compounds can enhance the abundance of beneficial bacteria in the gut, thereby promoting gut health and immune function (Zhang et al., 2021). In contrast, single-flavored TCM focuses more on the effects of specific ingredients and often performs well in regulating certain specific flora. For example, some single-flavored TCM has been proven to be effective in preventing and treating liver fibrosis by regulating intestinal tracts. This is achieved by the composition of the bacterial flora (Liu et al., 2021).

Secondly, TCM extracts and TCM active ingredients extract specific ingredients from Chinese medicine to enhance their biological activity. TCM extracts usually retain a variety of active ingredients, so they have a comprehensive effect in regulating GM and can improve intestinal metabolism and inflammation (Gao et al., 2023). The active ingredients of TCM are single compounds, and studies have shown that their effects on specific GM are more direct and clear. For example, some active ingredients can regulate the levels of bacterial metabolites, thereby improving the intestinal environment (He et al., 2023).

Judging from the similarities, all these forms of TCM promote intestinal health by modulating the composition of the GM and enhancing the abundance of beneficial flora. Research shows that the mechanism of action of TCM is closely related to diet, and both have similar effects in regulating GM. In addition, research in recent years has increasingly emphasized the interaction between TCM and GM, believing that the anti-tumor effect can be improved by regulating these flora (Zhang et al., 2021; Wei et al., 2024).

In general, TCM has shown significant potential in regulating GM. Different forms of TCM have their own characteristics and advantages. Future research can further explore their interactions and the possibility of comprehensive application. In order to provide more effective solutions for intestinal health.

### 4.2 Mechanisms of microbiota-gut-organ axis regulation by traditional Chinese medicine

The application of genomics technology is crucial in analyzing the roles and relationships between TCMs, GM, and the host. This technology can provide new insights and ideas for studying the efficacy and mechanism of action of TCMs, as well as help elucidate the functions and molecular mechanisms of GM. In recent years, the development of histological technologies and the application of multi-omics systems in TCMs have shown that changes in the structure and function of GM are caused by the interaction between TCMs and GM. This interaction activates a variety of signaling pathways within the microbiota-gut-organ axis and provides a new opportunity to understand the molecular mechanism of targeting GM with TCMs to treat diseases in the extraintestinal distal system.

#### 4.2.1 microbiota-gut-brain axis

The microbiota-gut-brain axis (MGB) is a two-way information exchange system between the gut and the brain. It includes GM and is an important pathway for integrating and regulating brain and gut functions. In recent years, some scientists have suggested that the concept of “heart” in Chinese medicine may include certain functions of the “brain” in modern medicine, while the small intestine may include certain functions. The concept of “heart and small intestine” in TCM encapsulates the intricate physiological link between the cerebrum (which is metaphorically referred to as the “heart” in TCM) and the intestines. This notion embodies the holistic approach inherent in TCM, where bodily functions are interconnected and interdependent. This concept can be extended to understand the relationship between the central nervous system and the GM. In a similar manner, TCMs can regulate the intestinal microecological environment by modulating GM, which can help alleviate the pathology of central nervous system disorders for therapeutic purposes.

Research on MGB suggests that GM is becoming a new target for TCMs in the treatment of central nervous system diseases, including insomnia, anxiety, depression, Alzheimer’s disease, and stroke (Li et al., 2023c; Ma et al., 2023). Ischemic stroke is a leading cause of death and disability in humans. Studies have shown that the traditional recipe San Hua Tang has a positive therapeutic effect on ischemic stroke by modulating GM and its metabolites SCFAs, promoting the expression of intestinal mucosal barrier tight junction proteins, and promoting the transition of microglia into the M2 condition, which eases inflammatory responses and reduces the extent of neurological damage and cerebral infarction in rats with ischemic stroke. However, the therapeutic effects were not observed in the groups treated with antibiotics alone or in combination with San Hua Tang and antibiotics. This suggests that GM is a key component in the therapeutic effect of San Hua Tang, with flora metabolites likely being its therapeutic target (Luo et al., 2023).

Baihe Jizihuang Tang, also called Baihe Jizi decoction, recorded in the TCM as “synopsis of the Golden Chamber,” is widely used to treat nervous system disorders caused by “Baihe disease” (TCM disease name, related to mental disorders). The prescription contains two botanical drugs, lily (mainly from the dry bulb of *Lilium lancifolium Thunb*.) and hen egg yolk, which can also be used as food. Studies have shown that the prescription can improve depressive behavior caused by chronic unpredictable mild stress (CUMS). These results suggest that these TCMs may improve depressive symptoms by regulating GM, primarily through restoring the balance of GM and its metabolites. Correlation analysis revealed that the restored GM levels were associated with changes in the levels of neurotransmitters such as 5-HT, neurotrophic factor (BDNF), and SCFAs. Additionally, TCMs can influence neurotransmitter metabolism, improve peripheral blood flow, and reduce neuroinflammation. This suggests that they can effectively alleviate depressive behavior caused by CUMS (Li et al., 2018; Zhu et al., 2021; Zhang X. et al., 2023). A study further confirmed, through fecal microbiota transplantation experiments, that TCMs may have an antidepressant effect based on the MGB axis. This effect may be related to signaling pathways such as PI3K/AKT/TLR4/NF- κ B and ERK/CREB/BDNF (Zhang Y. et al., 2023). TCMs have enormous potential for treating depression based on MGB.

Recent research has revealed a phenylalanine-tyrosine-dopamine synthesis pathway in intestinal bacterial cells. Furthermore, GM has been found to serve as a new source of dopamine *in vivo* (Wang et al., 2021b). Transplanting *Enterococcus faecalis* into mice with Parkinson’s disease has been shown to significantly increase dopamine levels in the brain and improve Parkinson’s symptoms. In addition, the combination of bacteria with berberine demonstrated a more significant therapeutic effect compared to berberine alone. Further studies have demonstrated that berberine can enhance the activity of tyrosine hydroxylase and facilitate the production of levodopa by intestinal bacteria through the promotion of dihydrobiopterin to tetrahydrobiopterin conversion. Moreover, certain GM such as *Escherichia coli*, *Lactobacillus*, *Streptococcus*, *Enterococcus* and *Bacillus* have the ability to synthesize and release neurotransmitters and neuromodulators that significantly influence brain development and behavior. These findings offer valuable insights into the use of MGB-based TCMs for the treatment of neurological disorders (Strandwitz, 2018).

**FIGURE 3:**
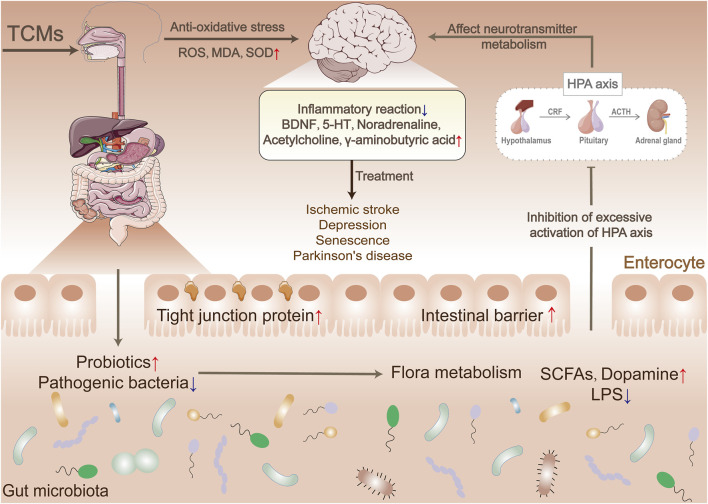
Mechanisms of microbiota-gut-brain axis regulation by traditional Chinese medicines.

TCMs can effectively target GM and its metabolites to regulate the gut-brain axis, mediate the regulation of HPA, influence the synthesis and release of neurotransmitters, reduce inflammatory responses, and regulate the central nervous system through the activation of associated signaling pathways (Figure 3). However, the current understanding of the regulatory mechanism of TCMs on MGB is limited, and further in-depth studies are needed to reveal the details and potential mechanisms.

#### 4.2.2 Microbiota-gut-liver axis

In modern medicine, there is increasing recognition of the intrinsic anatomical and functional connection between the liver and intestines. They have the ability to communicate with each other in both directions via the bile ducts, the portal vein, and the circulatory system (Bauer et al., 2022). For example, the liver transports various substances, including bile salts, antimicrobial molecules (such as IgA), and liver products (such as inflammatory mediators, acetaldehyde, butyric acid, and oxidized trimethylamine), through the biliary tract to the intestinal lumen. This affects the composition of intestinal fluid and the integrity of the intestinal barrier. Of these substances, bile salts play a crucial role as signaling molecules that can regulate a range of metabolic processes in the liver through receptors such as farnesoid X receptor (FXR). Additionally, intestinal factors, particularly microbial metabolites such as acetaldehyde, ethanol, and butyric acid, can enter the liver via the portal vein and influence liver function while regulating metabolic processes in the liver.

The bidirectional relationship between the intestine and liver in metabolic regulation has received significant attention in recent years, particularly in terms of how TCMs impact the occurrence and progression of diseases such as liver fibrosis and liver cancer through their influence on the GM. Studies have shown that supplementing with the probiotic L. rhamnosus can reduce liver inflammation and fibrosis, indicating a potential association between GM composition and these conditions (Jayakumar and Loomba, 2019). TCMs can influence the occurrence and development of liver fibrosis by regulating the GM, inhibiting intestinal barrier dysfunction, and reducing inflammatory responses. For instance, ursolic acid has been shown to enhance the abundance of *Lactobacillus* and Bifidobacterium in the GM, improve bacterial malnutrition, promote the stability of the GM, and inhibit the progression of liver fibrosis. Schisandra chinensis (Turcz.) Baill. has been found to safeguard liver health and prevent heart failure by modulating the GM and restoring the abnormal bile acid profile (Li H. et al., 2020). Furthermore, specific TCMs have the ability to inhibit the activation of hepatic stellate cells, enhance liver function, and consequently impede the progression of liver fibrosis by activating signaling pathways such as TGF-β/Smad and TLR4/MyD88/NF-κB (Liu et al., 2021).

Bile acids are vital components of bile and play a crucial role in both intestinal and liver signaling pathways. Studies have demonstrated that Xiayuxue decoction, a TCM formula, composing of three natural medicines: *Rheum officinale* Baill., *Prunus persica* (L.) Batsch and *Eupolyphaga sinensis* Walker, can enhance the production of bile salt hydrolase by increasing the population of *Bacteroides* and *Lactobacillus* in the intestines of rats with hepatocellular carcinoma. As a result, there is an elevation in primary bile acid levels, which subsequently stimulates interferon-gamma production by NKT cells in the liver, thereby exerting an anti-cancer effect. Utilizing bile acids as messengers to modulate the immune response of CXCR6+ NKT cells in the liver represents a novel therapeutic strategy for treating hepatocellular carcinoma, as supported by additional research findings (Deng et al., 2024). Furthermore, the TCM salidroside has been shown to ameliorate nonalcoholic steatohepatitis through its impact on the gut microbiota-bile acid-FXR axis. Its mechanism involves regulating the stability of the GM, enhancing lipid deposition, and reducing inflammatory damage in the livers of mice with nonalcoholic steatohepatitis, resulting in significant reductions in nonalcoholic steatohepatitis severity and liver triglyceride levels. Additionally, it activates FXR by modulating bile acid metabolism, particularly with respect to TaMCA, TβMCA, and βCDCA (Li M. et al., 2020).

Research shows that maintaining intestinal homeostasis and preserving intestinal barrier function are crucial regulatory factors in the progression from nonalcoholic fatty liver disease to more advanced stages. A study conducted by Yu et al. (2022) revealed the potential of the active metabolite Schisantherin A, found in Schisandra chinensis (Turcz.) Baill., to restore intestinal barrier function, enhance intestinal permeability, suppress LPS-TLR4 signaling, decrease lipopolysaccharide release, and alleviate intestinal inflammation. These findings suggest that the use of Schisantherin A can effectively enhance the occurrence and progression of non-alcoholic fatty liver disease induced by a high-fat diet.

**FIGURE 4:**
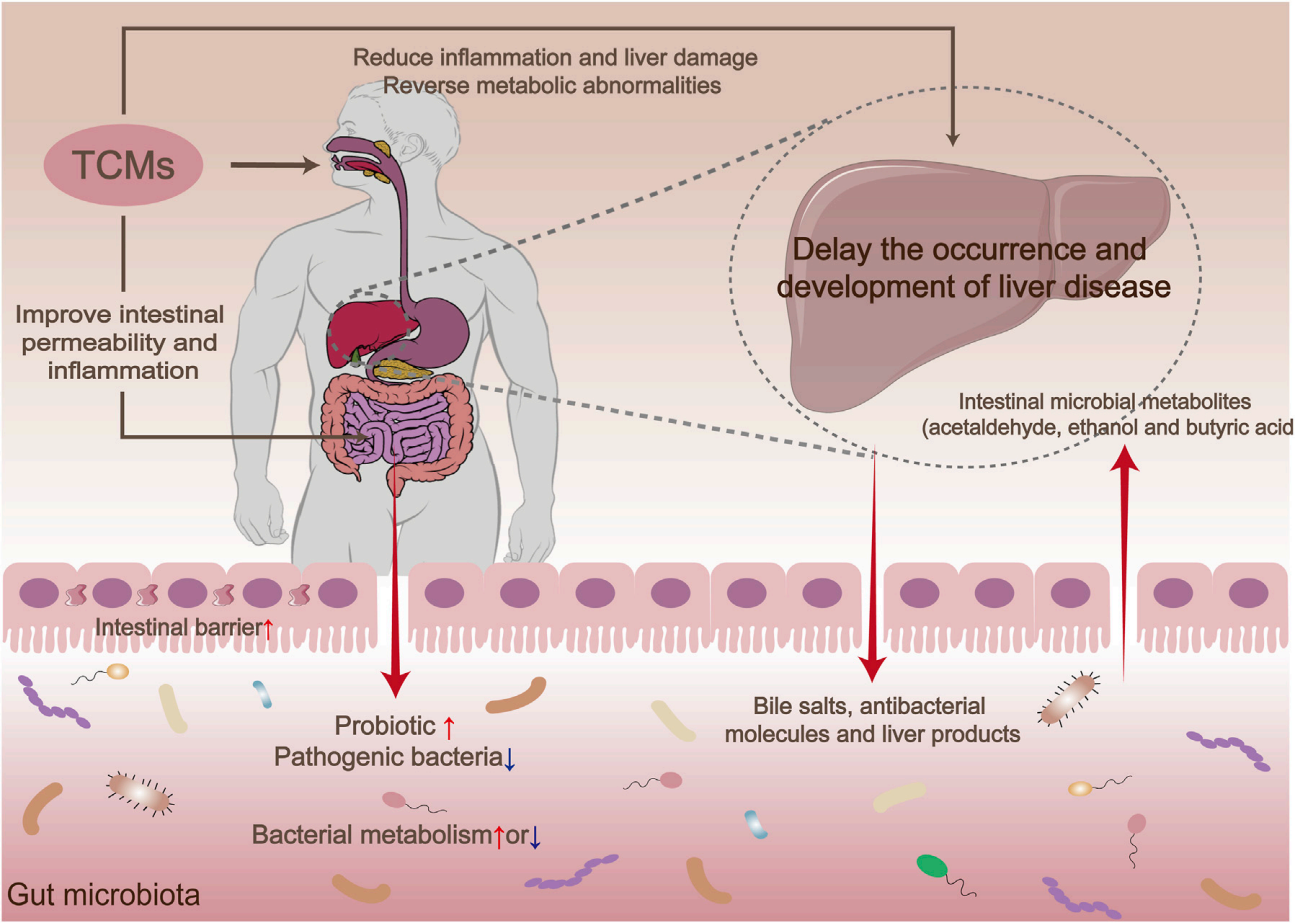
Mechanisms of microbiota-gut-liver axis regulation by traditional Chinese medicines.

TCMs regulate liver function and can delay the progression of liver diseases by influencing different aspects of the microbiota-gut-liver axis. These effects encompass the regulation of gut microbial composition, modulation of gut-liver signaling, suppression of inflammatory responses, and the protection of hepatocytes (Figure 4).

#### 4.2.3 Microbiota-gut-lung axis

The microbiota-gut-lung (MGL) axis represents a reciprocal regulatory pathway involving the GM and its metabolites. This pathway influences the physiological state of the gut and lungs by modulating immune response signaling pathways, as well as altering the activity of T lymphocyte subsets and inflammatory signals. Studies have demonstrated a close association between alterations in the structure of the GM and the development of various lung diseases. For example, disruptions in the GM caused by antibiotics can increase the body’s susceptibility to pneumonia and Th2 cell-induced asthma (Hill and Artis, 2013). A study was conducted to investigate the therapeutic effects of a botanical drug metabolite on allergic asthma combined with dysbiosis in rats. The study employed three treatment methods: lung treatment, intestinal treatment, and lung-intestinal combination treatment. The results showed that the lung-intestinal combination treatment was more effective than intestinal or lung treatment alone in restoring lung function and the GM in the model rats (Wen et al., 2017). These findings provide further evidence of the therapeutic interaction between the intestines and the lungs.

As a traditional medical system, TCMs play a crucial role in maintaining the microbial balance of the gut and lungs. It has been found that TCMs can regulate the microbial community in the gut, which in turn influences lung health. For example, Chinese botanical drug formulas such as Qi Bai Ping Lung Capsule (QBPF) and Xuan Bai Cheng Qi Tang (XBCQ) improved lung function in chronic obstructive pulmonary disease (COPD) rats by increasing the relative abundance of probiotics (e.g., *Akkermansia*) and pathogens (e.g., S*treptococcus*) bacteria that are significantly associated with inflammatory biomarkers (e.g., TNF-α, IL-1β, and MMP-9) (Wang et al., 2021c; Jia et al., 2022).

There is mounting evidence that the GM and its metabolites, SCFAs, play a crucial role in establishing and regulating the pulmonary immune system in both mice and human. Furthermore, the deletion of regulatory T (Treg) cells in the intestinal lamina propria affects the composition of the GM, while maintaining the balance between Th17 and Treg cells is vital in regulating the pathogenesis of chronic obstructive pulmonary disease (COPD) (Kehrmann et al., 2020). The results of the study indicate a significant positive correlation between the administration of probiotics influenced by QBPF and XBCQ and improvements in body weight and lung function. Conversely, there was a negative correlation observed with Th17/Treg ratios and pro-inflammatory cytokines. These findings suggest a strong association between the GM and the pulmonary immune mechanism. However, further substantiation is required by using aseptic or antibiotic-treated animal models in combination with colony transplantation. Targeting the GM to modulate the host immune response is an important mechanism for TCMs to treat lung-related diseases. Studies have demonstrated the effectiveness of TCMs such as Astragalus mongholicus Bunge, Shaoyao-Gancao Tang, and Qinbaiqingfei concentrate pills in regulating the immune function of the lung-intestinal mucosa and improving conditions such as acute lung tissue damage, asthma, and *mycoplasma* pneumonia (Cui et al., 2018; Liu et al., 2022; He et al., 2023).

Furthermore, studies have demonstrated that microbial metabolites, specifically SCFAs, have a positive impact on immune regulation. The Gu Ben Fang Xiao decoction, an experiential formula proposed by Professor Yuren Jiang, was found to reverse intestinal dysbiosis in asthmatic mice and promote the growth of SCFAs-producing bacteria, including *Firmicutes*, *Lachnospiraceae*, and *Bifidobacteriaceae*. As a result, there was a substantial rise in SCFAs levels in the intestinal circulation, with acetate being particularly prominent. Moreover, additional experiments involving antibiotic mixtures and SCFA supplementation have demonstrated that acetate salts can significantly enhance the differentiation of regulatory T cells and stimulate a systemic immune response (Dong et al., 2020).

*Houttuynia cordata Thunb.* polysaccharides have been proven to improve the immune and intestinal barriers. This is achieved by inhibiting the release of inflammatory cytokines and the expression of TLR4-NF-κB in the lung, increasing the number of intestinal cuprocytes and increasing the secretion of sIgA and the expression of tight junction proteins. This improved the survival rate of mice infected with the influenza A virus H1N1 (Shi et al., 2022). Recent studies have found a close connection between GM and the antiviral effects of *H. cordata Thunb.* polysaccharides. The interaction between the two downregulates the migration of CCR6Th17/CCR6Treg cells from the intestinal mucosa-associated lymphoid tissues to the lung, which in turn regulates the Th17/Treg balance between the intestine and the lung. The Chinese botanical drug formulas Qingfeiyin decoction and Xuanbai-Chengqi decoction can also show better preventive and therapeutic effects on influenza A virus by remodeling flora homeostasis and inflammatory signaling pathways such as MAPK, TNFα, JAK-STAT and TLR7/MyD88/NF-кB downregulate (Huo et al., 2022; Li S. et al., 2022).

**FIGURE 5:**
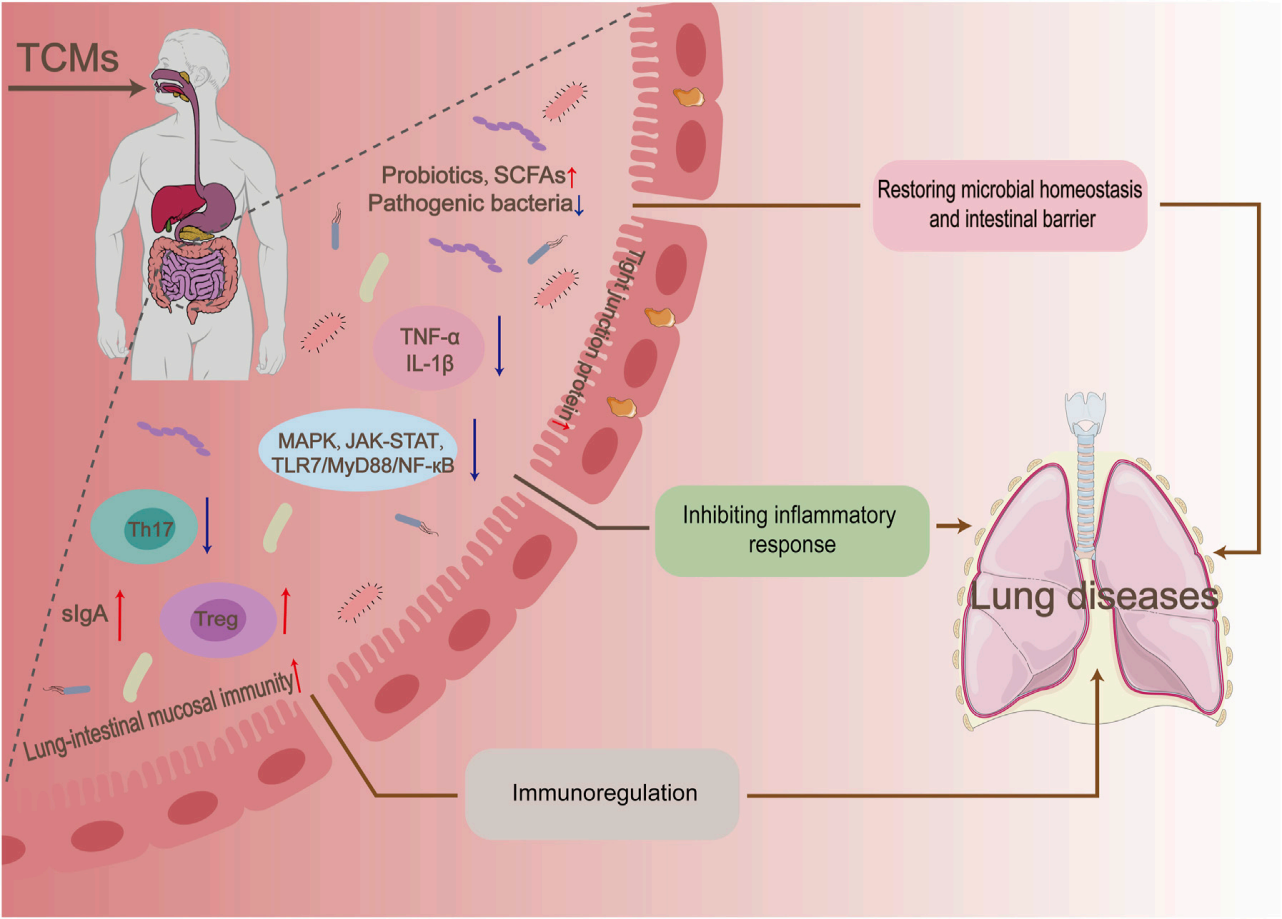
Mechanisms of microbiota-gut-lung axis regulation by traditional Chinese medicines.

TCMs regulate MGL interactions through multiple mechanisms, including the modulation of the GM and its metabolites, repair of the intestinal mucosal barrier, immune regulation, and modulation of inflammatory signaling pathways (Figure 5). While this field of study is still in its early stages, it holds great importance in deepening our understanding of the role of TCMs in regulating microbial homeostasis in the intestines and lungs, as well as in the treatment of related diseases.

To sum up, TCMs modulate the GM and affects the functions of the gut-brain axis, gut-liver axis and gut-lung axis, thereby improving the body’s health at multiple levels. This holistic treatment concept emphasizes the importance of the GM in maintaining physiological balance and treating disease processes. With the in-depth study of GM in modern medicine, the application prospects of TCM in this field have become increasingly broad, providing modern medicine with new ideas and methods to deal with increasingly complex health challenges.

## 5 Discussion and perspectives

The exploration of the modulatory effects of TCMs on the GM and the Microbiota-Gut-X Axis is a captivating field with significant implications. The GM’s diverse roles in promoting health, and the emerging concept of the gut-organ axis, provide a rich backdrop for understanding the potential of TCMs (Sun et al., 2023).

The evidence presented in this review highlighting the efficacy of TCMs in regulating the gastrointestinal system and their possible connections with the GM is encouraging. However, it is important to recognize the limitations. One limitation is the complexity and heterogeneity of TCM formulations, which make it challenging to precisely attribute specific effects to individual components (Luo et al., 2021; Zhou et al., 2021). Additionally, the lack of standardized methods for studying TCM-GM interactions can lead to inconsistent results (Yang et al., 2022).

Future research needs and priorities should focus on several key areas. Firstly, more in-depth mechanistic studies are required to comprehensively understand how TCMs interact with the GM and the Microbiota-Gut-X Axis. This may involve advanced molecular and cellular techniques. Secondly, large-scale clinical trials with well-defined endpoints are essential to validate the therapeutic potential of TCMs in modulating the GM and its impact on various diseases. Longitudinal studies to monitor the long-term effects and stability of these modulations would also be valuable. Furthermore, integrating TCMs with modern omics technologies, such as metagenomics and metabolomics, can provide a more holistic understanding of their effects. Exploring the potential synergies or antagonisms between TCMs and other therapeutic modalities, such as probiotics or dietary interventions, is another important area for future exploration.

In terms of perspectives, the continued growth and understanding in this field hold great promise. The potential of TCMs to offer novel therapeutic approaches for various disorders related to the GM and the Microbiota-Gut-X Axis is an exciting prospect. However, interdisciplinary collaboration and rigorous scientific investigation will be crucial to unlocking the full potential and ensuring the validity and reliability of findings.
